# SVM Optimization for Brain Tumor Identification Using Infrared Spectroscopic Samples

**DOI:** 10.3390/s18124487

**Published:** 2018-12-18

**Authors:** Himar Fabelo, Samuel Ortega, Elizabeth Casselden, Jane Loh, Harry Bulstrode, Ardalan Zolnourian, Paul Grundy, Gustavo M. Callico, Diederik Bulters, Roberto Sarmiento

**Affiliations:** 1 Institute for Applied Microelectronics (IUMA), University of Las Palmas de Gran Canaria (ULPGC), Campus de Tafira, Las Palmas 35017, Spain; sortega@iuma.ulpgc.es (S.O.); gustavo@iuma.ulpgc.es (G.M.C.); roberto@iuma.ulpgc.es (R.S.); 2 Wessex Neurological Centre, University Hospital Southampton, Tremona Road, Southampton SO16 6YD, UK; lizziecasselden@doctors.net.uk (E.C.); janeloh@nhs.net (J.L.); ardalan.zolnourian@uhs.nhs.uk (A.Z.); paul.grundy@uhs.nhs.uk (P.G.); dbulters@nhs.net (D.B.); 3 Department of Neurosurgery, Addenbrookes Hospital and University of Cambridge, Cambridge CB2 0QQ, UK; hb252@cam.ac.uk

**Keywords:** spectroscopy, tissue diagnostics, medical imaging, support vector machines, brain cancer

## Abstract

The work presented in this paper is focused on the use of spectroscopy to identify the type of tissue of human brain samples employing support vector machine classifiers. Two different spectrometers were used to acquire infrared spectroscopic signatures in the wavenumber range between 1200–3500 cm^−1^. An extensive analysis was performed to find the optimal configuration for a support vector machine classifier and determine the most relevant regions of the spectra for this particular application. The results demonstrate that the developed algorithm is robust enough to classify the infrared spectroscopic data of human brain tissue at three different discrimination levels.

## 1. Introduction

Currently, brain tumor diagnosis mainly relies on the histopathological analysis of tissue biopsies, where the expert decision, based on visual inspection of the tissue slides, is the gold standard for diagnosis. In some cases, this expert decision system can be biased depending on the experience of the pathologist in the diagnosis of brain samples. In 2016, the World Health Organization (WHO) published the last Classification of Tumors of the CNS (Central Nervous System), where the diagnosis of the tissue is performed through employing molecular analysis, in addition to traditional histopathological analysis [1]. In this sense, the use of new techniques that could help in the automatic identification of brain tissue will improve the reliability of the diagnosis.

Optical techniques have been extensively investigated on various tissues as an aid tool for classifying tumor type and grade [2,3]. Infrared (IR) spectroscopy measures the absorption or transmission intensity against wavenumber (different wavenumbers excite different covalent bonds) [4]. Therefore, altered molecular make-up is demonstrated through differences in intensity between various types of tissues. Based on epithelial thickening, altered nuclear/cytoplasmic ratio, dysplasia, and neovascularization, IR spectroscopic differences offer non-invasive, cost-effective and rapid methods for screening [2,5]. In prepared tissue sections on slides, IR absorption spectroscopy has demonstrated great potential to differentiate between tumor grades and tissue types [6,7] without exogenous fluorophores. This is achieved based on differences in lipid, protein, and nucleic acid contents, which are altered when the tissue becomes malignant [6]. The best correlation to malignancy is thought to be the lipid content [6,8]. Differences are demonstrated in cell cultures and tissue, and have been tenuously associated with increased cell motility, in line with chromatography and H&E (hematoxylin and eosin) staining [6,7,8,9].

The work exposed in this paper employs an IR spectroscopic dataset of brain tissue specimens for the development of a decision support system for tissue diagnosis based on a supervised machine learning algorithm. The support vector machines (SVMs) are supervised learning algorithms that are aimed at finding the maximum margin hyperplane that separates two classes [10]. In this first stage, where the hyperplane is identified (also called the training stage), previously labeled data are used to generate the SVM model. It is possible to classify new data by determining on which side of the hyperplane they are located, and hence establish their class membership. In case the samples are not linearly separable, the use of kernel functions is required to map the input space into a higher-dimensional feature space where the data can be separated. These kernel functions have specific parameters that can be tuned in order to obtain the best discrimination among the different classes. The characteristics of the data to be classified and the type of the classification problem will determine the optimal kernel and parameters to be used. SVMs combined with medical spectroscopy samples have been widely used to analyze different types of diseases, such us the tissue characterization or the diagnostics of lymph nodes in breast cancer [11,12], the diagnosis of skin cancer in mice [13], or the analysis of blood samples to detect dengue infection [14], among others. SVMs have been also used to classify the identify samples of primary tumors of brain metastases obtained using Raman spectroscopy [15]. In this work, the authors achieved an accuracy of 96.7% in the discrimination of tumor, necrosis, and normal brain. On the other hand, IR spectroscopic samples of brain tissue have been analyzed by Dreissig et al. [16] using partial least squares (PLS) regression to identify different types of lipids that could improve the diagnosis of brain tumors. Decision tree-based algorithms, such as Random Forest, combined with a two-dimensional (2D) correlation analysis have been also employed to diagnose brain cancer from serum samples using IR spectroscopy [17]. In this study, 433 patients were involved, collecting nine spectra from each in the range between 600–4000 cm^−1^. The results obtained were quite promising, achieving sensitivities and specificities in the discrimination of cancer and non-cancer serum samples up to 92.8% and 91.5%, respectively.

Although several algorithms have been employed in the literature to analyze spectroscopic samples, in general, SVMs are preferred over other classification algorithms in different fields due to their strong theoretical foundations, good generalization capabilities, and ability to find optimal solutions when a limited number of training samples are available [10,18,19]. In addition, SVMs provide good performance when the training datasets are highly unbalanced [20,21]. For these reasons, SVMs have been employed in this work, due to the available IR spectroscopic dataset being highly unbalanced and having only a limited number of samples.

Four different SVM kernels have been analyzed, optimizing their hyperparameters (kernel parameters whose values are set prior to the learning process) for the specific application of distinguishing the IR spectroscopic samples of brain tissue using only their spectral information as input features for the classifier. The type of tissue (tumor or normal), the grade of primary tumors (grade II, grade III, and grade IV), and the type of normal tissue (mixed normal, grey matter, and white matter) have been classified, finding the optimal SVM configuration (kernel and hyperparameters) to classify this type of sample. Finally, the most relevant regions in the IR spectra have been determined and analyzed employing the optimal classifier configuration.

## 2. Materials and Methods

The experiments accomplished in this research work were performed employing IR spectroscopic data from human brain samples processed using a supervised machine learning algorithm in order to find the most suitable configuration to accurately classify the data. The following sections will describe the methodology and the materials employed to reach the goal of distinguishing different brain tissue samples using spectroscopy.

### 2.1. Brain Samples

Patients undergoing craniotomy at the Wessex Neurological Centre of the University Hospital of Southampton, United Kingdom (UK), were consented prior to surgery. Patients either had a preliminary diagnosis of glioma or were undergoing a lobectomy for epilepsy treatment. The IR spectroscopic data were collected in two different acquisition campaigns. Ethical approvals were granted under REC08/H0505/165 and REC14/SC/0108 for the first data acquisition campaign (DC1) and for the second data acquisition campaign (DC2), respectively. The normal brain tissue and tumor tissue that were employed in this experiment were resected and not used for diagnostic purposes. Brain tissue was not removed in excess to that required for patients’ treatment. In DC1, a total of 23 patients were recruited over eight months. After refining the method to create pellets that are appropriate for spectroscopy, the samples were obtained. In three cases, some overlying cortex for analysis was obtained; two of these samples had clear grey and white matter. In DC2, a total of eight patients were recruited over six months, and samples were collected during surgery. In two cases, patients were epileptic and were scheduled for a lobectomy. The remaining cases consisted of a mixture of high-grade and low-grade gliomas, where mainly samples of overlying normal cortex were obtained for analysis, due to the lack of normal samples in DC1.

Tissue samples were identified as tumor by the operating surgeon, who was assisted by image guidance. En bloc resection specimens were collected, along with the tissue derived from the ultrasonic aspirator system, which is used extensively in developing a plane around the tumor, and debulking the tumor mass. Samples were washed in sterile 0.9% saline to remove visible blood traces where appropriate. Then, they were weighed and air-dried at approximately 40 °C until they reached a consistency compatible with grinding. Once samples were dried, they were ground into a homogenous powder with KBr powder, using a pestle and mortar. Then, the resulting mixture was fractionated into 0.5-g portions and pressed at ~10 tons in a pellet press with a vacuum facility, to create a solid pellet that is suitable for mounting in the spectrometer. Samples were stored individually at −20 °C with a sachet of silica gel in order to reduce water absorption by KBr.

### 2.2. Spectrographic Acquisition Systems

The two data acquisition campaigns were recorded using two different spectrometers. In DC1, data were collected through an FT-IR (Fourier transform infrared) spectrometer, Varian 600-IR (Varian Inc., Palo Alto, CA, USA), set in transmission mode and assembled in Agilent Resolutions Pro V.5 (Agilent Technologies, Santa Clara, CA, USA) software, where data were transformed into absorption spectra. This spectrometer covers the wavenumber range from 400 cm^−1^ to 6000 cm^−1^ with a spectral resolution of 1.93 cm^−1^, obtaining 2906 spectral bands. Background was recorded at 20 scans/minute, and each of the sample pellets were recorded at 15 scans/min, which were all obtained at a resolution of 4 cm^−1^. The spectrometer was continuously purged with nitrogen during its use, and a new background spectrum was recorded at regular intervals. DC2 was captured using a different FT-IR spectrometer, Spectrum BX FT-IR Spectrometer (PerkinElmer, Waltham, MA, USA), set in absorption mode and collected by Spectrum v5.3.1 software (PerkinElmer, Waltham, MA, USA). This spectrometer obtains the samples in the wavenumber rage from 1000 cm^−1^ to 4000 cm^−1^ with a spectral resolution of 2 cm^−1^, having 1501 spectral bands. The previously described acquisition procedure was also followed in this data campaign.

Table 1 shows the total number of IR spectroscopic samples collected in both data campaigns, specifying the type of tissue of each sample. Twenty-three patients from DC1 and eight patients from DC2 were included in this study, collecting a total of 246 spectral signatures. These two data campaigns were merged in order to generate a unique dataset where the samples of the two different spectrometers were included. Three different discrimination levels (DLs) were established to distinguish the different types of tissue available in the database. The use of these DLs implies the definition of different scenarios where the classification of IR spectroscopic data is accomplished by using different abstraction levels for tissue description, and hence, defining a hierarchical labeling where the classification is performed. Discrimination level 1 (DL1) distinguishes between tumor and normal samples. Discrimination level 2 (DL2) allows the discrimination between the grades of the tumor samples (grade II, grade III, and grade IV) and normal samples. Finally, discrimination level 3 (DL3) distinguishes between the different grades of the tumor samples and the different types of normal samples (grey matter, white matter, and mixed normal).

### 2.3. Data Pre-Processing

The spectroscopic signatures obtained during both acquisition data campaigns were denoised and normalized employing a smooth filter (five-point moving average), and a normalization process was independently applied to each spectroscopic signature using MATLAB^®^ (The MathWorks Inc., Natick, MA, USA). The normalization was applied in order to fit the spectral signatures between zero and one, thus isolating the samples from different illumination conditions. Since the two data campaigns were captured using different spectrometers, the spectroscopic samples were spectrally adapted in order to merge both datasets. The spectral signatures from DC1 were split to obtain spectroscopic samples covering the range from 1000 cm^−1^ to 4000 cm^−1^, which is the range of the spectrometer employed in the DC2. Since the spectral resolution of both datasets were different (1.93 cm^−1^ for DC1 and 2 cm^−1^ for DC2), a cubic spline interpolation was performed to the DC1 samples in order to fit the DC1 data along the spectral range of DC2 [22]. Finally, the extreme bands of the spectroscopic samples (from 1000 cm^−1^ to 1200 cm^−1^ and from 3500 cm^−1^ to 4000 cm^−1^) were removed in order to avoid the noise introduced by the sensor of the DC2 dataset in such bands. After the pre-processing steps, both datasets were merged in a single dataset composed by spectroscopic signatures that cover the wavenumber rage from 1200 cm^−1^ to 3500 cm^−1^ with a spectral resolution of two cm^−1^, which has 1151 spectral bands.

**Figure 1:**
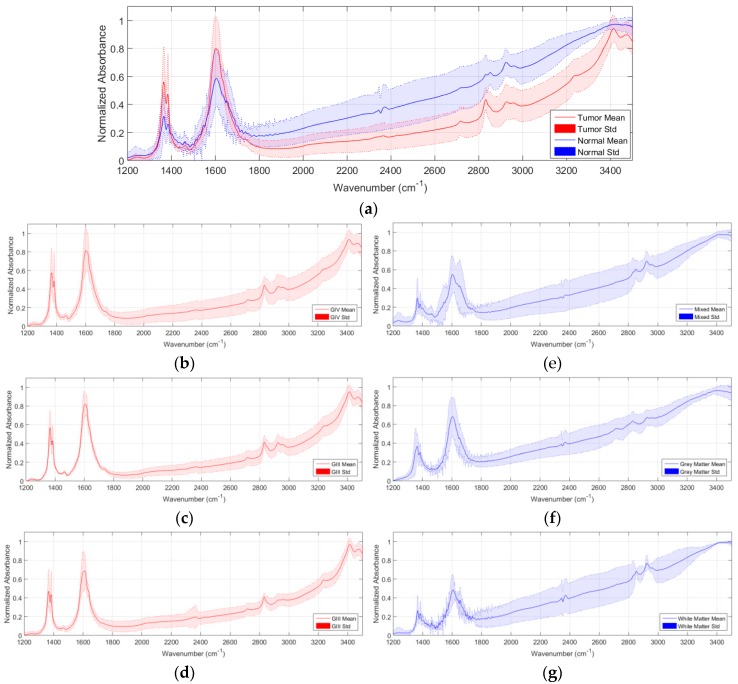
Average and standard deviation of the pre-processed spectroscopic signatures. (**a**) Tumor vs. normal samples; (**b**–**d**) Grade IV, III, and II tumor samples, respectively; (**e**–**g**) Mixed, grey matter, and white matter normal samples, respectively.

Figure 1a shows the average and standard deviation (STD) of the pre-processed spectral signatures of tumor and normal samples that compose the IR spectroscopic database. As it can be seen in this figure, there are several wavenumber ranges where the differences between the normal samples and the tumor samples are evident. As stated in [6], the region comprised between 1300–1500 cm^−1^ is mainly affected by the deformation vibration of alkyl groups CH_2_ and CH_3_, while the range 1500–1800 cm^−1^ is associated to the *C* = 0 stretching and N–H bending vibrations of the amide groups (amide I and II respectively), comprising the peptide linkages of proteins. Furthermore, in the wavenumber region between 2800–3000 cm^−1^, the spectra are dominated by acyl chain stretching vibrations with fatty acids of lipids as the main contributors. These regions will be taken into account in Section 3 for an optimization of the classification system, where only these regions of interest will be employed to train the system and classify the spectroscopic samples. In Figure 1b–d, the average and standard deviation of the pre-processed spectroscopic signatures of grade IV (GIV), grade III (GIII), and grade II (GII) tumor samples are shown respectively. Finally, Figure 1e–g show the average and standard deviation of the pre-processed spectroscopic signatures of mixed, grey matter, and white matter normal samples.

### 2.4. Support Vector Machines for IR Spectroscopic Samples Classification

The experiments that were performed in this study to classify the IR spectroscopic samples of brain tumor were carried out using an SVM classifier. LIBSVM was employed as the classifier implementation [23] using Matlab^®^ environment. Since SVMs have different hyperparameters that can be fine-tuned to improve the outcomes of the classification, an extensive optimization analysis was performed. In this study, the classification results obtained with four different SVM kernels were compared. Linear, polynomial, radial basis function (RBF) and sigmoid kernels were tested. Polynomial, RBF, and sigmoid kernels were analyzed in order to find the optimal parameters that provide the best classification results for this particular case. All of the kernels have one common parameter called cost (*C*). This parameter is the constant of constraint violation that observes whether a data sample is classified on the wrong side of the decision limit. The specific parameters to tune up each kernel are detailed herewith. In the RBF kernel, the width of the Gaussian radial basis function can be adjusted by the parameter *γ*. In the sigmoid kernel, the parameters that can be adjusted are the slope (*γ*) and the intercept constant (*cf*). Finally, polynomial kernel employs the parameters *γ*, *cf*, and *d*, which are the coefficient of the polynomial function, the coadditive constant and the degree of the polynomial, respectively. Table 2 presents the mathematical expressions of each kernel.

**Figure 2:**
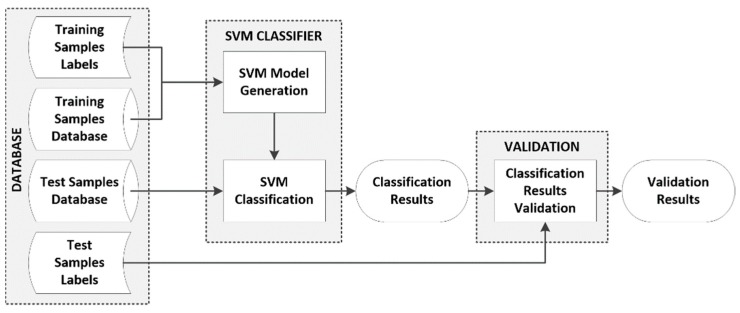
Block diagram of the classification framework.

Figure 2 shows the SVM classification framework that was employed in this work to optimize the parameters of the SVM kernels and obtain the classification accuracy results. The database is composed by the spectroscopic samples that have been captured and labeled, identifying the type of tissue (tumor or normal), the grade of the tumor tissue (grade II, grade III, and grade IV) and the type of normal tissue (mixed normal, grey matter, and white matter). This database was split into test and training datasets, following a repeated fivefold cross-validation method, to generate the SVM model and obtain the classification results. The results presented in this paper are the average values obtained in 10 repetitions of the fivefold cross-validation.

### 2.5. Evaluation Metrics

The results provided by the classification systems were evaluated using standard metrics to this end: sensitivity, specificity, and overall accuracy (ACC). These metrics are commonly used as statistical measures of the performance of binary classification methods [24,25,26,27]. Sensitivity is related to the test’s ability to identify a condition correctly. It is obtained as the number of true positives (TP) divided by the total number of true positives and false negatives (FN) in a population (Equation (1)). Specificity is related to the test’s ability to exclude a condition correctly. It is obtained as the number of true negatives (TN) divided by the total number of true negatives and false positives (FP) in a population (Equation (2)). Finally, ACC is calculated by dividing the total number of successful results by the total population (Equation (3)). The other metrics that were also computed for this study are detailed in Section S1 of the Supplementary Material.

(1) $$Sensitivity=\frac{TP}{TP+FN}$$

(2) $$Specificity=\frac{TN}{TN+FP}$$

(3) $$ACC=\frac{TP+TN}{TP+FP+FN+TN}$$

## 3. Experimental Results and Discussion

In this section, the results obtained during the hyperparameter optimization and the study related to the selection of the most suitable wavenumber region to distinguish efficiently the different types of tissue are shown and discussed.

### 3.1. SVM Parameter Optimization

**Figure 3:**
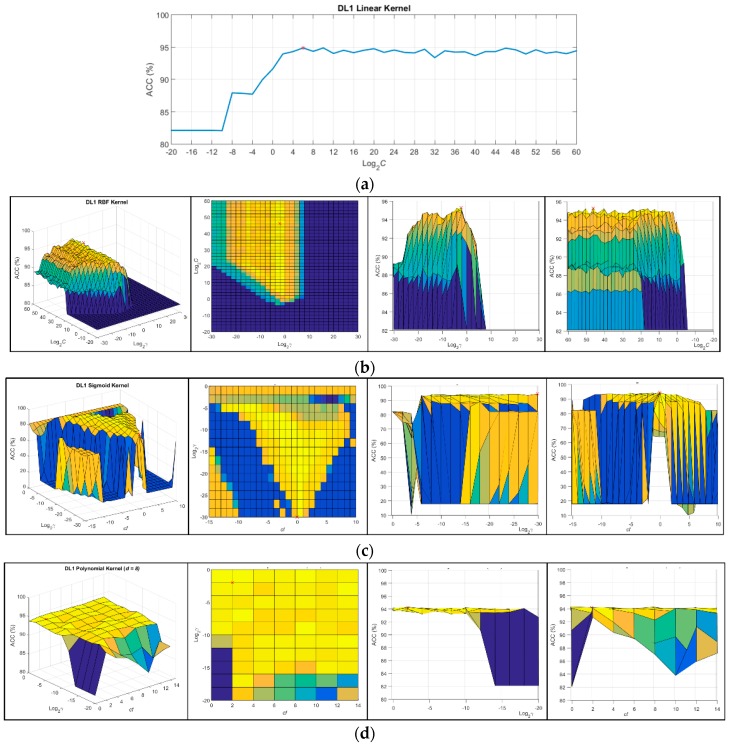
Grid search representation of the ACC results obtained in the coarse tuning for each kernel employing the DL1. (**a**) Linear kernel results; (**b**) RBF kernel results; (**c**) Sigmoid kernel results; (**d**) Polynomial kernel results with a fixed degree parameter (*d* = 8).

**Figure 4:**
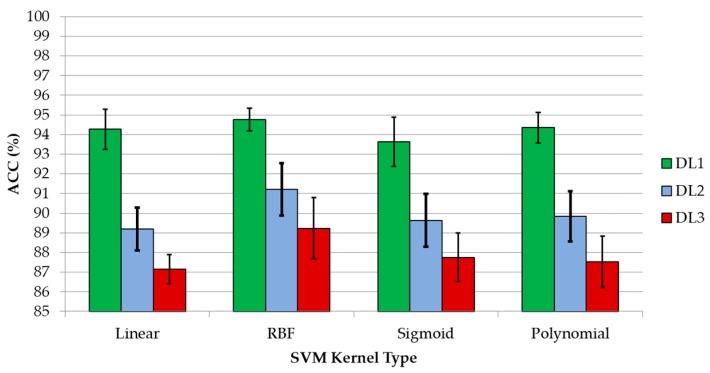
Average and standard deviation of the overall accuracy results achieved with the optimal hyperparameters for each kernel type and discrimination level.

Due to the presence of parameters in the SVM configuration that can be adjusted in order to obtain the optimal performance in the classification results, an exhaustive analysis of these parameter values were performed for each discrimination level of the entire dataset (DL1, DL2, and DL3). To optimize the values of the parameters for each kernel, a repeated cross-validation was performed. The parameters were modified with a certain step between the limits presented in Table 3 for each DL and type of kernel. These limits were determined empirically after several analyses of the parameter limits, performing a coarse grid search, in which the average ACC values of the cross-validation process were inspected. Figure 3 shows an example of the grid search performed for DL1 per each type of kernel. The grid search of the linear kernel shows the ACC results versus the logarithmic values of parameter *C* (Figure 3a). Figure 3b shows the RBF kernel grid search where the representation of the parameters *C* versus *γ* show the ACC value for each *C* − γ pair. In case of a sigmoid kernel, parameter *C* is fixed with the previously calculated value in the RBF grid search, and the best pair of γ − *cf* parameters was identified (Figure 3c). Finally, in the polynomial kernel, the parameter *C* was fixed with the previously optimized value obtained in the RBF kernel grid search, and each γ − *cf* pair was computed for each *d* value. At that point, the best three parameters (γ, *cf*, and *d*) that optimize the ACC result were searched. Figure 3d shows the grid search that was performed to find of the best pair of γ − *cf* parameters for a certain degree value (*d* = 8 in this case). The grid search was performed in two steps. First, a coarse grid search was performed, and then, a fine grid search was done in order to find the more accurate optimal parameters. In Figure 4, a graphical representation of the average values of the ACC results that were obtained with each fine-tuned kernel for each discrimination level is presented. As it can be seen, the RBF kernel is the one that offers the best results.

However, computing the sensitivity and specificity metrics is also required in order to validate whether the RBF kernel is the one that offers the best tradeoff between sensitivity and specificity. To compute this using the optimal parameters identified for each kernel, a classification of each discrimination level was performed to obtain the sensitivity and specificity values. The classification was performed employing the one-versus-all method, where the metric values for a certain class are calculated, taking into account the remaining classes as one single class [28,29].

**Figure 5:**
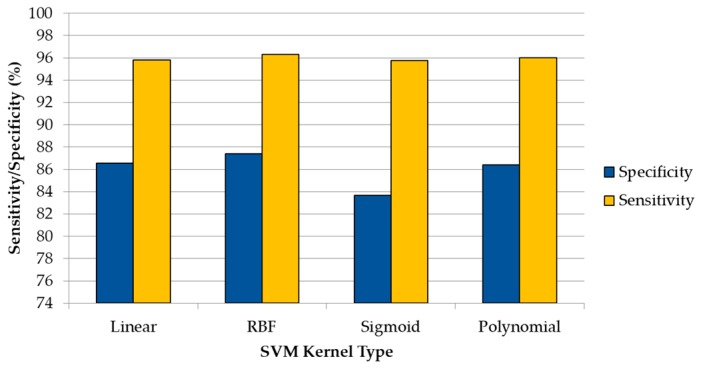
Average sensitivity and specificity results obtained (using the 10 × 5 cross-validation) with the optimal hyperparameters for each kernel type in the DL1 (tumor vs. normal).

Figure 5 shows the average sensitivity and specificity results that were obtained employing the spectroscopic dataset with the DL1, where the goal is to discriminate between tumor and normal samples. Although all of the kernels provide accurate results with slight differences, the RBF kernel obtains the best values for this DL. Nevertheless, all of the classifiers are able to achieve competitive results. Furthermore, as it can be seen in the results, the sensitivity of the classifier (the ability to correctly classify the tumor samples) is higher than the specificity (the ability to correctly classify the normal samples), which is mainly due to the low number of normal samples (44 in DL1) with respect to the number of tumor samples (202 in DL1). A significant increment in the number of normal samples in the database could probably provide better specificity results, increasing also the ACC of the system. In addition to these results, Table S1 provides the detailed mean and standard deviation classification results, where it is possible to observe that the precision of the classifier to identify the tumor samples accurately is quite high (above 96.5% for all of the kernels), being a reliable method to determine the histopathological diagnosis of brain tissues by employing a spectrographic system.

**Figure 6:**
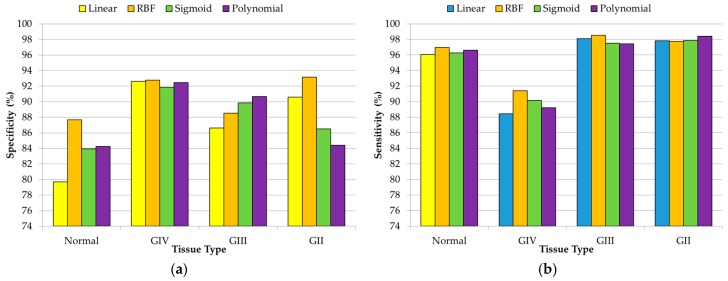
Average specificity (**a**) and sensitivity (**b**) results obtained (using the 10 × 5 cross-validation) with the optimal hyperparameters for each kernel type in the DL2 (normal vs. grade IV vs. grade III vs. grade II).

In the case of the DL2 dataset, the specificity and sensitivity results are obtained using the one-versus-all technique to obtain the multiclass classification results to distinguish between different grades of tumor samples (grade VI, grade III, and grade II) and normal samples. In Figure 6, the average specificity and sensitivity results that were achieved in this multiclass classification for each kernel type are shown. Although the polynomial kernel offers the best results for the specificity of GIII and sensitivity of GII tissue types, in general, RBF provided the best classification results, obtaining an overall accuracy of 91.22%. The grade IV class obtained the best results in terms of precision (94.49%) and F-score (93.60%) with respect to the other classes (see Table S2). In the same way as in the DL1 results, this precision difference is mainly produced due to the highly unbalanced number of samples that belongs to each class (147 samples for the grade IV class and less than 44 samples for each of the remaining classes).

**Figure 7:**
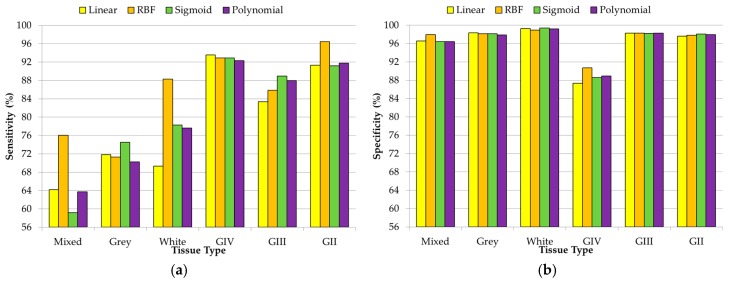
Average specificity (**a**) and sensitivity (**b**) results obtained (using the 10 × 5 cross-validation) with the optimal hyperparameters for each kernel type in the DL3 (normal mixed vs. grey matter vs. white matter vs. grade IV vs. grade III vs. grade II).

Finally, Figure 7 shows the results obtained for the classification of the DL3 dataset, where all of the different types of normal tissue (mixed, grey matter, and white matter) and tumor tissue (grade IV, grade III, and grade II) were classified. In this case, there is not a unique kernel that provides the best results. However, the RBF kernel provides the best overall accuracy results (89.22%), achieving the most balanced precision and F-score results for all of the tissue types (see Table S3). Although this DL offers lower accuracy than the one obtained in both DL1 and DL2, it still offers competitive results for discriminating the different tissue types. In the same way as DL2, the higher precision was obtained with the grade IV tissue samples (94.35%) due to the higher amount of samples that belong to this class. However, as can be seen in Figure 7a, in the case of normal samples, all of the sensitivity results are lower than the sensitivity values that are achieved for the tumor samples. This is mainly produced due to the lower number of normal tissue samples (10 samples of mixed normal, 18 samples of grey matter, and 16 samples of white matter). An increment in each type of normal sample could improve the sensitivity results in the discrimination between the different types of normal samples.

### 3.2. Wavenumber Region Optimization

**Figure 8:**
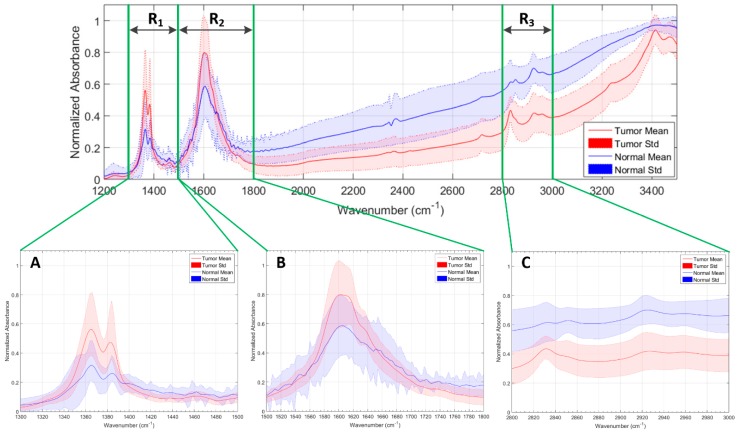
Relevant wavenumber regions selected for the SVM analysis. (**A**) R_1_: region from 1300 cm^−1^ to 1500 cm^−1^; (**B**) R_2_: region from 1500 cm^−1^ to 1800 cm^−1^; (**C**) R_3_: region from 2800 cm^−1^ to 3000 cm^−1^.

As previously described in Section 2.3, in [6], three different relevant wavenumber regions were analyzed and identified for IR spectroscopic samples of brain tissue within the range between 1200–3500 cm^−1^. In this paper, these regions have been evaluated employing the SVM classifier with the RBF kernel in order to determine the wavenumber region that offers the best accuracy results using the minimum number of spectral features. Table 4 details the wavenumber regions and the number of spectral bands of each dataset that were employed in this study. In addition, Figure 8 graphically represents the partition of the spectral signatures in the aforementioned three different wavenumber regions (R_1_, R_2_, and R_3_). One region, R_123_, has been defined comprising the union between R_1_, R_2_, and R_3_.

**Figure 9:**
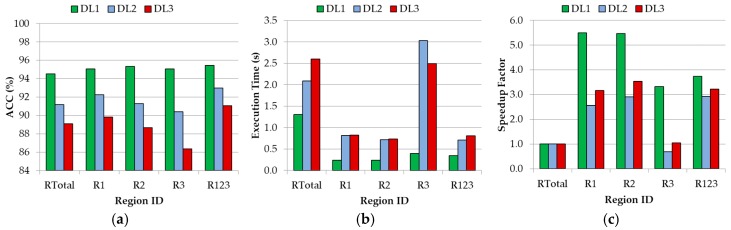
Average of the overall accuracy results (**a**), execution time (**b**), and speedup factor (respect to the R_Total_ execution time) achieved with the SVM RBF kernel with the optimal hyperparameters for each DL, using the different wavenumber regions.

The previously described wavenumber regions were analyzed employing the SVM classifier using the RBF kernel with its optimal parameters for each discrimination level (DL1: $C=2^{46.55}/\gamma =2^{-2.25}$; DL2: $C=2^{21.1}/\gamma =2^{-3.6}$; and DL3: $C=2^{49.05}/\gamma =2^{-3.25}$). Figure 9a shows the accuracy results achieved for each discrimination level when using each wavenumber region that was presented in Table 4. As can be seen in the results, the use of the R_123_ dataset (that involves the wavenumber regions $R_{1}\cup R_{2}\cup R_{3}$) offers the best accuracy results for each DL (DL1: 95.44%; DL2: 92.97%; DL3: 91.06%), improving the results obtained using the complete wavenumber region (R_Total_). In addition, this reduction of spectral bands in the dataset provides an important decrease in the execution time of the algorithm (Figure 9b). A speedup of ~3× was obtained employing the R_123_ dataset with respect to the time required for the R_Total_ dataset execution (Figure 9c). These time results were obtained employing the LIBSVM [23] in Matlab^®^ using an Intel^®^ Core™ i7-4790K at 4.00 GHz. Tables S4–S6 show the detailed average classification results, which were computed employing each wavenumber region for each DL and their respective execution times. It is worth noticing that when the R_3_ region is used, a high execution time is obtained with respect to the other regions (especially for DL2 and DL3). This is caused because the training of the algorithm requires more time to converge, producing also lower accuracy results.

In the case of DL1 (Table S4), regions R_1_, R_2_, and R_3_ provide improved accuracy results with respect to the R_Total_ wavenumber region. In addition, the use of these regions allows obtaining a reduced execution time, especially in R_1_ and R_2_ (speedup of 5.5×). In the case of DL2 (Table S5) and DL3 (Table S6), the optimal values for each metric are distributed among the different wavenumber regions. However, the wavenumber region R_123_ is the region that offers the best results in terms of accuracy compared with the R_Total_ dataset.

In conclusion, the use of the combination of the three wavenumber regions shown in Figure 4 ($R_{1}\cup R_{2}\cup R_{3}$) has proved to be the best range to identify the different brain tissue types (in the three DLs). Furthermore, the use of this conjunction of wavenumber regions implies a significant reduction in the execution time of the algorithm compared with the use of the full wavenumber region.

## 4. Conclusions

The use of IR spectroscopy for the identification of brain tissue samples is an emerging technique that may offer, in the near future, a reliable diagnostic tool to complement the traditional histopathological diagnosis. The work that is presented in this paper uses two different FT-IR spectrometers to obtain spectroscopic signatures of brain tissue samples in the wavenumber range from 1200 cm^−1^ to 3500 cm^−1^. Samples were obtained from 31 different patients during two different data acquisition campaigns, collecting 246 spectral signatures that were labeled according to their corresponding histopathological diagnosis. These spectroscopic signatures were employed to develop and optimize a machine-learning model based on an SVM classifier. Four different SVM kernels were evaluated to find the optimal configuration parameters for classifying three discrimination levels of brain tissue types (DL1: tumor versus normal; DL2: grade IV versus grade III versus grade II versus normal; DL3: grade IV versus grade III versus grade II versus mixed normal versus grey matter versus white matter). The results were obtained by averaging the overall accuracy results of an iterative cross-validation.

One of the main conclusions of this study is that, having available an extensive training dataset for the generation of the supervised machine learning algorithm, it is possible to accurately predict the type of brain tissue through exclusively using its IR spectral features. Results with an accuracy above 87% were achieved with all of the SVM kernels for the three DLs. The optimal SVM configuration for each DL employs the RBF kernel, but using different hyperparameters in each DL. The optimal hyperparameters for DL1 were $C=2^{46.55}$ and $\gamma =2^{-2.25}$, achieving an ACC value of 94.76%, while the optimal configuration for DL2 was $C=2^{27.1}$ and $\gamma =2^{-3.6}$, achieving 91.22% of ACC. Finally, in DL3, the best ACC result (89.22%) was obtained with the parameters $C=2^{49.05}$ and $\gamma =2^{-3.25}$. The inclusion of other evaluation metrics that take into account the performance per class has reinforced the validity of the results, demonstrating that the RBF kernel is the most suitable SVM kernel for this application.

After the determination of the optimal configuration of the SVM classifier, three different wavenumber regions were studied, concluding that searching for the optimal spectral features within the spectral range can improve both the accuracy and the execution time of the classification. The combination of the regions covering from 1300 cm^−1^ to 1800 cm^−1^ and 2800 cm^−1^ to 3000 cm^−1^ (formed by 352 spectral bands) obtained the best accuracy results for each DL (DL1: 95.44%; DL2: 92.97%; DL3: 91.06%). Furthermore, an average speed up of 3× was achieved in the execution of the algorithm compared with the use of the complete spectroscopic signatures dataset.

Another remarkable result that was obtained from this study shows that it is possible to successfully exploit the IR spectroscopic data that was captured using different spectrometers by applying the appropriate data processing. Unfortunately, due the unbalanced amount of the sample types from each spectrometer, it was not possible to evaluate each one independently. Although promising results were achieved in this work, a larger IR spectroscopic database, involving more quantity of each type of sample, could lead to a more generic SVM model. Having enough samples of different types of primary tumors (glioblastoma, oligodendroglioma, astrocytoma, etc.) and different types of secondary tumors (lung, renal, breast, etc.) could allow the generation of a model that identifies the grade and type (primary) or origin (secondary) of the tumor tissue in all of the cases.

## Figures and Tables

**Table 1:** Infrared (IR) spectroscopic samples database of human brain tissue. DL1: discrimination level 1, DL2: discrimination level 2, DL3: discrimination level 3.

Type of Tissue			#Total Samples			#Patients
DL1	DL2	DL3	DL1	DL2	DL3	
Tumor	Grade IV		202	147	147	17
	Grade III			30	30	4
	Grade II			25	25	3
Normal	Grey Matter		44	44	18	6
	White Matter				10	4
	Mixed				16	2
Total:			246			31

**Table 2:** Mathematical expressions of the support vector machine (SVM) kernels. RBF: radial basis function.

Kernel	Formula	Hyperparameters
Linear	*k*(*x*,*y*) = *x^T^* · *y*	*C*
RBF	*k*(*x*,*y*) = *exp*(−*γ* · ‖*x* − *y*‖^2^)	*C*, *γ*
Sigmoid	*k*(*x*,*y*) = *tanh*(*γ* · *x^T^* · *y* + *cf*)	*C*, *γ*, *cf*
Polynomial	*k*(*x*,*y*) = (*γ* · *x^T^* · *y* + *cf*)^*d*^	*C*, *γ*, *cf*, *d*

**Table 3:** Results of the hyperparameter tuning analysis for each discrimination level and SVM kernel. ACC: accuracy.

Discrimination Level	Kernel	Parameter	Coarse Grid Search		Fine Grid Search		ACC (STD) (%)
			Initial/Step/Final	Optimal	Initial/Step/Final	Optimal	
DL1	Linear	*C*	$2^{-20}/2^{2}/2^{60}$	$2^{6}$	$2^{5}/2^{0.05}/2^{7}$	$2^{5.35}$	94.27 (±1.02)
	RBF	*C*	$2^{-20}/2^{2}/2^{60}$	$2^{46}$	$2^{45}/2^{0.05}/2^{47}$	$2^{46.55}$	94.76 (±0.5)
		γ	$2^{-30}/2^{2}/2^{30}$	$2^{-2}$	$2^{-3}/2^{0.05}/2^{-1}$	$2^{-2.25}$	
	Sigmoid	*C*	2^46.55^ (fixed)				93.62 (±1.25)
		γ	$2^{-30}/2^{2}/2^{0}$	$2^{-30}$	$2^{-31}/2^{0.05}/2^{-29}$	$2^{-29}$	
		cf	−15/1/10	0	−1/0.05/1	0	
	Polynomial	*C*	2^46.55^ (fixed)				94.35 (±0.78)
		γ	$2^{-20}/2^{2}/2^{0}$	$2^{-2}$	$2^{-3}/2^{0.05}/2^{-1}$	$2^{-1.1}$	
		cf	0/2/14	2	1/0.05/3	2.65	
		d	0/2/14	8	7/0.2/9	7	
DL2	Linear	*C*	$2^{-20}/2^{2}/2^{60}$	$2^{34}$	$2^{33}/2^{0.05}/2^{35}$	$2^{33}$	89.19 (±1.09)
	RBF	*C*	$2^{-20}/2^{2}/2^{60}$	$2^{28}$	$2^{27}/2^{0.05}/2^{29}$	$2^{27.1}$	91.22 (±1.33)
		γ	$2^{-30}/2^{2}/2^{30}$	$2^{-4}$	$2^{-5}/2^{0.05}/2^{-3}$	$2^{-3.6}$	
	Sigmoid	*C*	2^27.1^ (fixed)				89.63 (±1.35)
		γ	$2^{-30}/2^{2}/2^{0}$	$2^{-8}$	$2^{-9}/2^{0.05}/2^{-7}$	$2^{-7.75}$	
		cf	−15/1/10	−3	−4/0.05/−2	−2.75	
	Polynomial	*C*	2^27.1^ (fixed)				89.83 (±1.27)
		γ	$2^{-20}/2^{2}/2^{0}$	$2^{-2}$	$2^{-3}/2^{0.05}/2^{-1}$	$2^{-1.35}$	
		cf	0/2/14	2	1/0.05/3	1.05	
		d	0/2/14	2	1/0.2/3	3	
DL3	Linear	*C*	$2^{-20}/2^{2}/2^{60}$	$2^{18}$	$2^{17}/2^{0.05}/2^{19}$	$2^{18.9}$	87.15 (±0.75)
	RBF	*C*	$2^{-20}/2^{2}/2^{60}$	$2^{50}$	$2^{49}/2^{0.05}/2^{51}$	$2^{49.05}$	89.22 (±1.56)
		γ	$2^{-30}/2^{2}/2^{30}$	$2^{-4}$	$2^{-5}/2^{0.05}/2^{-3}$	$2^{-3.25}$	
	Sigmoid	*C*	2^49.05^ (fixed)				87.75 (±1.24)
		γ	$2^{-30}/2^{2}/2^{0}$	$2^{-12}$	$2^{-13}/2^{0.05}/2^{-11}$	$2^{-11.15}$	
		cf	−15/1/10	−1	−2/0.05/0	−1	
	Polynomial	*C*	2^49.05^ (fixed)				87.53 (±1.30)
		γ	$2^{-20}/2^{2}/2^{0}$	$2^{-6}$	$2^{-7}/2^{0.05}/2^{-5}$	$2^{-5.95}$	
		cf	0/2/14	12	11/0.05/13	11.3	
		d	0/2/14	4	3/0.2/5	3.2	

**Table 4:** Wavenumber regions of the IR spectroscopic samples of brain tissue to be analyzed by the SVM classifier using the RBF kernel.

Region ID	Wavenumber Region (cm^−1^)	#Spectral Bands
R_Total_	1200–3500	1151
R_1_	1300–1500	101
R_2_	1500–1800	150
R_3_	2800–3000	101
R_123_	$R_{1}\cup R_{2}\cup R_{3}$	352

## Supplementary Materials

The following are available online at http://www.mdpi.com/1424-8220/18/12/4487/s1, S1: Evaluation Metrics, Table S1: Classification results (mean and standard deviation) for the 10 × 5 cross-validation of the DL1 (Tumor vs. Normal), Table S2: Classification results (mean and standard deviation) for the 10 × 5 cross-validation of the DL2 (Normal vs. Grade IV vs. Grade III vs. Grade II), Table S3: Classification results (mean and standard deviation) for the 10 × 5 cross-validation of the DL3 (Normal Mixed vs. Grey Matter vs. White Matter vs. Grade IV vs. Grade III vs. Grade II), Table S4: Classification results (mean and standard deviation) for the 10 × 5 cross-validation of the DL1 (Tumor vs. Normal) employing different wavenumber regions using the RBF kernel, Table S5: Classification results (mean and standard deviation) for the 10 × 5 cross-validation of the DL2 (Normal vs. Grade IV vs. Grade III vs. Grade II) employing different wavenumber regions using the RBF kernel, Table S6: Classification results (mean and standard deviation) for the 10 × 5 cross-validation of the DL3 (Normal Mixed vs. Grey Matted vs. White Matter vs. Grade IV vs. Grade III vs. Grade II) employing different wavenumber regions using the RBF kernel.
