# Supplementary material for: SVM Optimization for Brain Tumor Identification Using Infrared Spectroscopic Samples
Source: Sensors (Basel). 2018 Dec 18;18(12):4487. doi: 10.3390/s18124487 (PMC6308411; doi:10.3390/s18124487)
Supplement: Supplementary file 1 [file sensors-18-04487-s001.zip › Supplementary Material.docx]

**SVM Optimization for Brain Tumor Identification using Infrared Spectroscopic Samples**

**Supplementary Material**

1. Evaluation Metrics

The results provided by the classification system were evaluated using standard metrics to this end: sensitivity, specificity, accuracy (ACC), false negative rate (FNR), false positive rate (FPR), precision and F-score (F_1_). These metrics are commonly used as statistical measures of the performance of binary classification methods. Sensitivity is related to the tests ability to identify a condition correctly. It is obtained as the number of true positives (TP) divided by the total number of true positives and false negatives (FN) in a population (Eq. 1). Specificity is related to the tests ability to exclude a condition correctly. It is obtained as the number of true negatives (TN) divided by the total number of true negatives and false positives (FP) in a population (Eq. 2). ACC is calculated by dividing the total number of successful results by the total population (Eq. 3). FNR and FPR are related to sensitivity and specificity values as exposed in Eq. 4 and Eq. 5, respectively. Finally, the precision and the F-score (that computes the harmonic mean of precision and sensitivity) are calculated as Eq. 6 and Eq. 7, respectively.

| $Sensitivity=\frac{TP}{TP+FN}$ | (1) |
| --- | --- |
| $Specificity=\frac{TN}{TN+FP}$ | (2) |
| $ACC=\frac{TP+TN}{TP+FP+FN+TN}$ | (3) |
| $FNR=1-\frac{TP}{TP+FN}=1-Sensitivity$ | (4) |
| $FPR=1-\frac{TN}{TN+FP}=1-Specificity$ | (5) |
| $Precision=\frac{TP}{TP+FP}$ | (6) |
| $F_{1}=\frac{2\times TP}{2\times TP+FP+FN}=2\times\frac{Precision\times Sesitivity}{Precision+Sensitivity}$ | (7) |

1. Classification results (mean and standard deviation) for the 10x5 cross-validation of the DL1 (Tumor vs. Normal).

|  | Kernel Type | | | |
| --- | --- | --- | --- | --- |
|  | Linear | RBF | Sigmoid | Polynomial |
| ACC (%) | 94.27 ± 1.02 | 94.76 ± 0.58 | 93.62 ± 1.25 | 94.35 ± 0.78 |
| Sensitivity (%) | 95.82 ± 1.04 | 96.28 ± 0.44 | 95.74 ± 1.12 | 96.00 ± 0.75 |
| Specificity (%) | 86.54 ± 2.03 | 87.42 ± 3.15 | 83.65 ± 4.52 | 86.39 ± 3.28 |
| FNR (%) | 4.18 ± 1.04 | 3.72 ± 0.44 | 4.26 ± 1.12 | 4.00 ± 0.75 |
| FPR (%) | 13.46 ± 2.03 | 12.58 ± 3.15 | 16.35 ± 4.52 | 13.61 ± 3.28 |
| F_1_ (%) | 96.54 ± 0.60 | 96.82 ± 0.37 | 96.13 ± 0.76 | 96.58 ± 0.47 |
| Precision (%) | 97.28 ± 0.42 | 97.38 ± 0.78 | 96.53 ± 1.09 | 97.18 ± 0.81 |

1. Classification results (mean and standard deviation) for the 10x5 cross-validation of the DL2 (Normal vs. Grade IV vs. Grade III vs. Grade II).

|  | | **Kernel Type** | | | |
| --- | --- | --- | --- | --- | --- |
|  |  | **Linear** | **RBF** | **Sigmoid** | **Polynomial** |
| **ACC (%)** | | 89.19 ± 1.09 | **91.22 ± 1.33** | 89.63 ± 1.35 | 89.83 ± 1.27 |
| **Sensitivity (%)** | **Normal** | 79.70 ± 3.98 | **87.68 ± 2.74** | 83.94 ± 2.64 | 84.24 ± 3.08 |
|  | **GIV** | 92.60 ± 1.35 | **92.76 ± 1.73** | 91.84 ± 1.47 | 92.44 ± 1.43 |
|  | **GIII** | 86.62 ± 4.43 | 88.52 ± 7.63 | 89.86 ± 5.92 | **90.66 ± 4.90** |
|  | **GII** | 90.58 ± 3.87 | **93.15 ± 4.46** | 86.52 ± 3.37 | 84.39 ± 4.63 |
| **Specificity (%)** | **Normal** | 96.07 ± 0.86 | **96.98 ± 0.41** | 96.28 ± 0.79 | 96.63 ± 0.95 |
|  | **GIV** | 88.45 ± 1.78 | **91.41 ± 2.27** | 90.17 ± 1.34 | 89.21 ± 1.80 |
|  | **GIII** | 98.08 ± 0.76 | **98.51 ± 0.80** | 97.51 ± 0.61 | 97.42 ± 0.68 |
|  | **GII** | 97.84 ± 0.45 | 97.75 ± 0.66 | 97.85 ± 0.70 | **98.42 ± 0.53** |
| **FNR (%)** | **Normal** | 20.30 ± 3.98 | **12.32 ± 2.74** | 16.06 ± 2.64 | 15.76 ± 3.08 |
|  | **GIV** | 7.40 ± 1.35 | **7.24 ± 1.73** | 8.16 ± 1.47 | 7.56 ± 1.43 |
|  | **GIII** | 13.38 ± 4.43 | 11.48 ± 7.63 | 10.14 ± 5.92 | **9.34 ± 4.90** |
|  | **GII** | 9.42 ± 3.87 | **6.85 ± 4.46** | 13.48 ± 3.37 | 15.61 ± 4.63 |
| **FPR (%)** | **Normal** | 3.93 ± 0.86 | **3.02 ± 0.41** | 3.72 ± 0.79 | 3.37 ± 0.95 |
|  | **GIV** | 11.55 ± 1.78 | **8.59 ± 2.27** | 9.83 ± 1.34 | 10.79 ± 1.80 |
|  | **GIII** | 1.92 ± 0.76 | **1.49 ± 0.80** | 2.49 ± 0.61 | 2.58 ± 0.68 |
|  | **GII** | 2.16 ± 0.45 | 2.25 ± 0.66 | 2.15 ± 0.70 | **1.58 ± 0.53** |
| **F_1_ (%)** | **Normal** | 81.21 ± 2.74 | **87.23 ± 1.76** | 83.85 ± 2.12 | 84.76 ± 2.41 |
|  | **GIV** | 92.59 ± 0.95 | **93.60 ± 1.09** | 92.84 ± 0.72 | 92.75 ± 1.17 |
|  | **GIII** | 86.88 ± 3.78 | **88.93 ± 3.93** | 86.39 ± 4.26 | 86.40 ± 3.94 |
|  | **GII** | 86.18 ± 2.07 | **86.70 ± 4.64** | 84.36 ± 4.38 | 85.67 ± 3.36 |
| **Precision (%)** | **Normal** | 82.95 ± 3.90 | **86.82 ± 1.79** | 83.86 ± 3.46 | 85.45 ± 4.18 |
|  | **GIV** | 92.59 ± 1.26 | **94.49 ± 1.68** | 93.88 ± 0.91 | 93.06 ± 1.23 |
|  | **GIII** | 87.33 ± 5.16 | **90.00 ± 5.44** | 83.33 ± 4.16 | 82.67 ± 4.66 |
|  | **GII** | 82.40 ± 3.86 | 81.20 ± 5.67 | 82.40 ± 5.72 | **87.20 ± 4.54** |

1. Classification results (mean and standard deviation) for the 10x5 cross-validation of the DL3 (Normal Mixed vs. Grey Matter vs. White Matter vs. Grade IV vs. Grade III vs. Grade II).

|  | | **Kernel Type** | | | |
| --- | --- | --- | --- | --- | --- |
|  |  | **Linear** | **RBF** | **Sigmoid** | **Polynomial** |
| **ACC (%)** | | 87.15 ± 0.75 | **89.22 ± 1.56** | 87.75 ± 1.24 | 87.53 ± 1.30 |
| **Sensitivity (%)** | **Mixed** | 64.22 ± 9.31 | **76.04 ± 9.91** | 59.16 ± 12.91 | 63.77 ± 6.96 |
|  | **Grey** | 71.84 ± 4.18 | 71.29 ± 5.94 | **74.51 ± 10.03** | 70.28 ± 7.57 |
|  | **White** | 69.35 ± 6.21 | **88.27 ± 12.55** | 78.25 ± 9.05 | 77.66 ± 15.39 |
|  | **GIV** | **93.58 ± 0.87** | 92.91 ± 1.21 | 92.89 ± 1.03 | 92.33 ± 0.85 |
|  | **GIII** | 83.33 ± 3.85 | 85.86 ± 2.37 | **88.93 ± 3.79** | 87.99 ± 3.92 |
|  | **GII** | 91.33 ± 3.85 | **96.43 ± 4.44** | 91.21 ± 4.12 | 91.78 ± 3.00 |
| **Specificity (%)** | **Mixed** | 96.60 ± 0.36 | **97.96 ± 0.68** | 96.41 ± 0.48 | 96.44 ± 0.59 |
|  | **Grey** | **98.34 ± 0.44** | 98.15 ± 0.58 | 98.16 ± 0.45 | 97.88 ± 0.58 |
|  | **White** | 99.28 ± 0.55 | 98.97 ± 0.37 | **99.42 ± 0.50** | 99.23 ± 0.52 |
|  | **GIV** | 87.35 ± 2.17 | **90.71 ± 2.15** | 88.58 ± 1.40 | 88.91 ± 2.27 |
|  | **GIII** | 98.28 ± 0.64 | 98.27 ± 0.65 | 98.24 ± 0.63 | **98.28 ± 0.55** |
|  | **GII** | 97.64 ± 0.70 | 97.84 ± 0.24 | **98.09 ± 0.51** | 97.99 ± 0.46 |
| **FNR (%)** | **Mixed** | 35.78 ± 9.31 | **23.96 ± 9.91** | 40.84 ± 12.91 | 36.23 ± 6.96 |
|  | **Grey** | 28.16 ± 4.18 | 28.71 ± 5.94 | **25.49 ± 10.03** | 29.72 ± 7.57 |
|  | **White** | 30.65 ± 6.21 | **11.73 ± 12.55** | 21.75 ± 9.05 | 22.34 ± 15.39 |
|  | **GIV** | **6.42 ± 0.87** | 7.09 ± 1.21 | 7.11 ± 1.03 | 7.67 ± 0.85 |
|  | **GIII** | 16.67 ± 3.85 | 14.14 ± 2.37 | **11.07 ± 3.79** | 12.01 ± 3.92 |
|  | **GII** | 8.67 ± 3.85 | **3.57 ± 4.44** | 8.79 ± 4.12 | 8.22 ± 3.00 |
| **FPR (%)** | **Mixed** | 3.40 ± 0.36 | **2.04 ± 0.68** | 3.59 ± 0.48 | 3.56 ± 0.59 |
|  | **Grey** | **1.66 ± 0.44** | 1.85 ± 0.58 | 1.84 ± 0.45 | 2.12 ± 0.58 |
|  | **White** | 0.72 ± 0.55 | 1.03 ± 0.37 | **0.58 ± 0.50** | 0.77 ± 0.52 |
|  | **GIV** | 12.65 ± 2.17 | **9.29 ± 2.15** | 11.42 ± 1.40 | 11.09 ± 2.27 |
|  | **GIII** | 1.72 ± 0.64 | 1.73 ± 0.65 | 1.76 ± 0.63 | **1.72 ± 0.55** |
|  | **GII** | 2.36 ± 0.70 | 2.16 ± 0.24 | **1.91 ± 0.51** | 2.01 ± 0.46 |
| **F_1_ (%)** | **Mixed** | 60.44 ± 5.08 | **75.22 ± 8.43** | 56.57 ± 8.71 | 59.03 ± 6.47 |
|  | **Grey** | 75.53 ± 3.44 | 73.74 ± 4.75 | **75.61 ± 5.05** | 71.62 ± 3.85 |
|  | **White** | 76.03 ± 6.88 | 82.26 ± 7.20 | **82.64 ± 8.70** | 80.21 ± 11.81 |
|  | **GIV** | 92.87 ± 1.00 | **93.62 ± 1.00** | 92.97 ± 0.44 | 92.82 ± 0.89 |
|  | **GIII** | 85.99 ± 2.82 | 87.18 ± 2.50 | **88.66 ± 1.52** | 88.42 ± 2.63 |
|  | **GII** | 85.75 ± 2.95 | **88.82 ± 2.48** | 87.77 ± 2.53 | 87.66 ± 2.60 |
| **Precision (%)** | **Mixed** | 57.65 ± 4.64 | **74.71 ± 8.34** | 54.71 ± 6.23 | 55.29 ± 7.44 |
|  | **Grey** | **80.00 ± 5.68** | 77.06 ± 7.57 | 77.65 ± 5.41 | 74.12 ± 7.44 |
|  | **White** | 85.00 ± 11.79 | 78.00 ± 7.89 | **88.00 ± 10.33** | 84.00 ± 10.75 |
|  | **GIV** | 92.18 ± 1.64 | **94.35 ± 1.40** | 93.06 ± 0.90 | 93.33 ± 1.56 |
|  | **GIII** | 89.00 ± 4.17 | 88.67 ± 4.22 | 88.67 ± 4.22 | **89.00 ± 3.53** |
|  | **GII** | 81.20 ± 5.98 | 82.40 ± 2.07 | **84.80 ± 4.13** | 84.00 ± 3.77 |

1. Classification results (mean and standard deviation) for the 10x5 cross-validation of the DL1 (Tumor vs. Normal) employing different wavenumber regions using the RBF kernel.

|  | **Wavenumber Region** | | | | |
| --- | --- | --- | --- | --- | --- |
|  | **R_Total_** | **R_1_** | **R_2_** | **R_3_** | **R_123_** |
| **ACC (%)** | 94.51 ± 1.02 | 95.08 ± 0.64 | 95.33 ± 0.81 | 95.08 ± 0.83 | **95.44 ± 0.37** |
| **Sensitivity (%)** | 96.18 ± 0.75 | **97.41 ± 0.58** | 97.27 ± 0.42 | 97.08 ± 0.54 | 96.90 ± 0.32 |
| **Specificity (%)** | 86.66 ± 4.77 | 85.14 ± 3.93 | 86.58 ± 3.07 | 86.05 ± 2.71 | **88.54 ± 1.76** |
| **FNR (%)** | 3.82 ± 0.75 | **2.59 ± 0.58** | 2.73 ± 0.42 | 2.92 ± 0.54 | 3.10 ± 0.32 |
| **FPR (%)** | 13.34 ± 4.77 | 14.86 ± 3.93 | 13.42 ± 3.07 | 13.95 ± 2.71 | **11.46 ± 1.76** |
| **F_1_ (%)** | 96.67 ± 0.63 | 96.99 ± 0.41 | 97.15 ± 0.50 | 97.00 ± 0.50 | **97.24 ± 0.23** |
| **Precision (%)** | 97.18 ± 1.14 | 96.58 ± 1.13 | 97.03 ± 0.74 | 96.93 ± 0.65 | **97.57 ± 0.43** |
| **Time (s) / Speedup** | 1.30 / 1x | **0.24 / 5.50x** | **0.24 / 5.50x** | 0.39 / 3.32x | 0.35 / 3.74x |

1. Classification results (mean and standard deviation) for the 10x5 cross-validation of the DL2 (Normal vs. Grade IV vs. Grade III vs. Grade II) employing different wavenumber regions using the RBF kernel.

|  | | **Wavenumber Region** | | | | |
| --- | --- | --- | --- | --- | --- | --- |
|  |  | **R_Total_** | **R_1_** | **R_2_** | **R_3_** | **R_123_** |
| **OA (%)** | | 91.17 ± 1.51 | 92.27 ± 1.29 | 91.30 ± 1.14 | 90.40 ± 0.96 | **92.97 ± 1.53** |
| **Sensitivity (%)** | **Normal** | **87.96 ± 2.19** | 83.24 ± 2.96 | 82.59 ± 3.96 | 82.09 ± 3.60 | 86.64 ± 2.70 |
|  | **GIV** | 92.58 ± 1.76 | **94.41 ± 1.13** | 93.58 ± 0.99 | 93.66 ± 1.68 | 94.28 ± 1.18 |
|  | **GIII** | 88.31 ± 4.81 | **100.00 ± 0.00** | 95.60 ± 2.39 | 92.41 ± 3.22 | 96.65 ± 7.38 |
|  | **GII** | 92.56 ± 5.78 | 88.88 ± 5.54 | 90.93 ± 5.59 | 85.04 ± 7.28 | **92.72 ± 3.28** |
| **Specificity (%)** | **Normal** | 97.12 ± 0.30 | 97.96 ± 0.74 | **97.99 ± 0.75** | 97.32 ± 1.09 | 97.58 ± 0.50 |
|  | **GIV** | 91.63 ± 1.97 | 91.24 ± 1.61 | 90.74 ± 2.32 | 91.64 ± 1.60 | **92.79 ± 2.04** |
|  | **GIII** | 98.45 ± 0.76 | 98.62 ± 0.31 | 97.94 ± 0.84 | 97.63 ± 0.74 | **98.97 ± 1.02** |
|  | **GII** | 97.48 ± 0.70 | 98.19 ± 0.59 | 97.84 ± 0.51 | 97.09 ± 0.59 | **98.30 ± 0.31** |
| **FNR (%)** | **Normal** | **12.04 ± 2.19** | 16.76 ± 2.96 | 17.41 ± 3.96 | 17.91 ± 3.60 | 13.36 ± 2.70 |
|  | **GIV** | 7.42 ± 1.76 | **5.59 ± 1.13** | 6.42 ± 0.99 | 6.34 ± 1.68 | 5.72 ± 1.18 |
|  | **GIII** | 11.69 ± 4.81 | **0.00 ± 0.00** | 4.40 ± 2.39 | 7.59 ± 3.22 | 3.35 ± 7.38 |
|  | **GII** | 7.44 ± 5.78 | 11.12 ± 5.54 | 9.07 ± 5.59 | 14.96 ± 7.28 | **7.28 ± 3.28** |
| **FPR (%)** | **Normal** | 2.88 ± 0.30 | 2.04 ± 0.74 | **2.01 ± 0.75** | 2.68 ± 1.09 | 2.42 ± 0.50 |
|  | **GIV** | 8.37 ± 1.97 | 8.76 ± 1.61 | 9.26 ± 2.32 | 8.36 ± 1.60 | **7.21 ± 2.04** |
|  | **GIII** | 1.55 ± 0.76 | 1.38 ± 0.31 | 2.06 ± 0.84 | 2.37 ± 0.74 | **1.03 ± 1.02** |
|  | **GII** | 2.52 ± 0.70 | 1.81 ± 0.59 | 2.16 ± 0.51 | 2.91 ± 0.59 | **1.70 ± 0.31** |
| **F_1_ (%)** | **Normal** | 87.71 ± 1.03 | 86.99 ± 2.72 | 86.67 ± 2.38 | 85.01 ± 2.42 | **87.93 ± 1.68** |
|  | **GIV** | 93.62 ± 1.18 | 94.31 ± 1.03 | 93.75 ± 0.84 | 94.23 ± 0.78 | **94.79 ± 1.12** |
|  | **GIII** | 88.92 ± 4.22 | **95.09 ± 1.16** | 90.44 ± 3.52 | 87.91 ± 3.28 | 94.75 ± 6.82 |
|  | **GII** | 85.06 ± 5.45 | 86.71 ± 4.42 | 86.16 ± 4.19 | 79.96 ± 5.47 | **88.98 ± 2.39** |
| **Precision (%)** | **Normal** | 87.50 ± 1.20 | 91.14 ± 3.29 | **91.36 ± 3.35** | 88.41 ± 4.84 | 89.32 ± 2.16 |
|  | **GIV** | 94.69 ± 1.31 | 94.22 ± 1.08 | 93.95 ± 1.74 | 94.83 ± 1.12 | **95.31 ± 1.34** |
|  | **GIII** | 89.67 ± 5.08 | 90.67 ± 2.11 | 86.00 ± 5.84 | 84.00 ± 5.16 | **93.00 ± 6.93** |
|  | **GII** | 78.80 ± 5.98 | 84.80 ± 4.92 | 82.00 ± 4.32 | 75.60 ± 5.15 | **85.60 ± 2.80** |
| **Time (s) / Speedup** | | 2.09 / 1x | 0.81 / 2.57x | 0.72 / 2.90x | 3.03 / 0.69 | **0.71 / 2.93x** |

1. Classification results (mean and standard deviation) for the 10x5 cross-validation of the DL3 (Normal Mixed vs. Grey Matted vs. White Matter vs. Grade IV vs. Grade III vs. Grade II) employing different wavenumber regions using the RBF kernel.

|  | | **Wavenumber Region** | | | | |
| --- | --- | --- | --- | --- | --- | --- |
|  |  | **R_Total_** | **R_1_** | **R_2_** | **R_3_** | **R_123_** |
| **OA (%)** | | 89.10 ± 1.66 | 89.84 ± 1.03 | 88.66 ± 1.74 | 86.35 ± 1.29 | **91.06 ± 1.90** |
| **Sensitivity (%)** | **Mixed** | 72.73 ± 4.69 | 70.05 ± 5.42 | 82.16 ± 7.63 | 55.41 ± 6.18 | **84.53 ± 4.33** |
|  | **Grey** | **72.20 ± 9.70** | 69.89 ± 5.68 | 62.13 ± 4.32 | 72.16 ± 3.33 | 69.44 ± 4.84 |
|  | **White** | **91.96 ± 10.42** | 76.30 ± 11.04 | 79.25 ± 13.36 | 67.30 ± 3.20 | 85.68 ± 8.71 |
|  | **GIV** | 92.40 ± 1.26 | **94.11 ± 1.96** | 92.93 ± 2.03 | 93.29 ± 1.54 | 93.66 ± 1.90 |
|  | **GIII** | 86.52 ± 4.93 | **100.00 ± 0.00** | 96.19 ± 2.94 | 88.93 ± 5.00 | 97.31 ± 3.45 |
|  | **GII** | **96.86 ± 4.49** | 90.62 ± 4.16 | 89.06 ± 8.44 | 83.20 ± 9.91 | 92.11 ± 4.72 |
| **Specificity (%)** | **Mixed** | 97.79 ± 0.63 | 98.07 ± 0.45 | 97.77 ± 0.95 | 96.09 ± 0.43 | **98.32 ± 0.22** |
|  | **Grey** | 97.88 ± 0.69 | 98.25 ± 0.43 | 98.60 ± 0.73 | **98.61 ± 0.65** | 98.36 ± 0.51 |
|  | **White** | 99.11 ± 0.27 | **99.12 ± 0.15** | 98.69 ± 0.20 | 98.94 ± 0.45 | 98.99 ± 0.30 |
|  | **GIV** | 91.76 ± 3.11 | 91.78 ± 1.26 | 90.47 ± 2.28 | 89.53 ± 1.75 | **92.13 ± 2.29** |
|  | **GIII** | 98.27 ± 0.99 | 98.48 ± 0.66 | 97.34 ± 0.92 | 97.55 ± 0.80 | **99.39 ± 0.76** |
|  | **GII** | 97.51 ± 0.66 | 97.58 ± 0.78 | 98.11 ± 0.32 | 96.57 ± 0.63 | **97.93 ± 0.87** |
| **FNR (%)** | **Mixed** | 27.27 ± 4.69 | 29.95 ± 5.42 | 17.84 ± 7.63 | 44.59 ± 6.18 | **15.47 ± 4.33** |
|  | **Grey** | **27.80 ± 9.70** | 30.11 ± 5.68 | 37.87 ± 4.32 | 27.84 ± 3.33 | 30.56 ± 4.84 |
|  | **White** | **8.04 ± 10.42** | 23.70 ± 11.04 | 20.75 ± 13.36 | 32.70 ± 3.20 | 14.32 ± 8.71 |
|  | **GIV** | 7.60 ± 1.26 | **5.89 ± 1.96** | 7.07 ± 2.03 | 6.71 ± 1.54 | 6.34 ± 1.90 |
|  | **GIII** | 13.48 ± 4.93 | **0.00 ± 0.00** | 3.81 ± 2.94 | 11.07 ± 5.00 | 2.69 ± 3.45 |
|  | **GII** | **3.14 ± 4.49** | 9.38 ± 4.16 | 10.94 ± 8.44 | 16.80 ± 9.91 | 7.89 ± 4.72 |
| **FPR (%)** | **Mixed** | 2.21 ± 0.63 | 1.93 ± 0.45 | 2.23 ± 0.95 | 3.91 ± 0.43 | **1.68 ± 0.22** |
|  | **Grey** | 2.12 ± 0.69 | 1.75 ± 0.43 | 1.40 ± 0.73 | **1.39 ± 0.65** | 1.64 ± 0.51 |
|  | **White** | 0.89 ± 0.27 | **0.88 ± 0.15** | 1.31 ± 0.20 | 1.06 ± 0.45 | 1.01 ± 0.30 |
|  | **GIV** | 8.24 ± 3.11 | 8.22 ± 1.26 | 9.53 ± 2.28 | 10.47 ± 1.75 | **7.87 ± 2.29** |
|  | **GIII** | 1.73 ± 0.99 | 1.52 ± 0.66 | 2.66 ± 0.92 | 2.45 ± 0.80 | **0.61 ± 0.76** |
|  | **GII** | 2.49 ± 0.66 | 2.42 ± 0.78 | 1.89 ± 0.32 | 3.43 ± 0.63 | **2.07 ± 0.87** |
| **F_1_ (%)** | **Mixed** | 72.41 ± 6.05 | 72.69 ± 4.23 | 76.25 ± 7.94 | 52.84 ± 4.14 | **81.48 ± 1.98** |
|  | **Grey** | 72.53 ± 8.14 | 73.71 ± 4.71 | 70.92 ± 5.54 | **77.27 ± 4.34** | 73.95 ± 4.52 |
|  | **White** | **85.86 ± 6.16** | 78.20 ± 5.65 | 75.03 ± 7.37 | 72.03 ± 4.88 | 81.33 ± 5.69 |
|  | **GIV** | 93.73 ± 1.38 | **94.56 ± 1.03** | 93.59 ± 1.35 | 93.67 ± 0.81 | 94.37 ± 1.41 |
|  | **GIII** | 87.45 ± 4.78 | 94.68 ± 2.52 | 88.59 ± 3.94 | 86.48 ± 4.18 | **96.61 ± 3.84** |
|  | **GII** | **87.22 ± 3.53** | 84.83 ± 4.52 | 86.70 ± 4.52 | 77.15 ± 5.62 | 87.06 ± 5.37 |
| **Precision (%)** | **Mixed** | 72.35 ± 8.34 | 75.88 ± 5.85 | 72.35 ± 12.10 | 51.18 ± 6.23 | **78.82 ± 3.04** |
|  | **Grey** | 73.53 ± 8.88 | 78.24 ± 5.58 | 82.94 ± 8.96 | **83.53 ± 7.74** | 79.41 ± 6.35 |
|  | **White** | 81.00 ± 5.68 | **81.00 ± 3.16** | 72.00 ± 4.22 | 78.00 ± 9.19 | 78.00 ± 6.32 |
|  | **GIV** | 95.10 ± 1.97 | 95.03 ± 0.85 | 94.29 ± 1.44 | 94.08 ± 1.16 | **95.10 ± 1.50** |
|  | **GIII** | 88.67 ± 6.70 | 90.00 ± 4.44 | 82.33 ± 6.30 | 84.33 ± 5.22 | **96.00 ± 4.92** |
|  | **GII** | 79.60 ± 5.48 | 80.00 ± 6.53 | **84.80 ± 2.53** | 72.40 ± 5.15 | 82.80 ± 7.32 |
| **Time (s) / Speedup** | | 2.60 / 1x | 0.82 / 3.17x | **0.74 / 3.54x** | 2.50 / 1.04x | 0.81 / 3.22x |
